# Coordinate transformation method for beam grazing angle calculation of space-based early warning radar

**DOI:** 10.1038/s41598-026-42233-4

**Published:** 2026-03-02

**Authors:** XiaoBin Huang, Yan Zhang

**Affiliations:** Air Force Early Warning Academy, Wuhan, China

**Keywords:** Space-based early warning radar, Beam grazing angle, Coordinate transformation, Orbital perturbation, Aerospace engineering, Electrical and electronic engineering

## Abstract

With the rapid development of space technology and the increasing demand for global security early warning, space-based early warning Radar (SBR) is playing an increasingly important role in defending against potential threats. Based on the perturbed motion model of low-earth orbit satellites, this paper proposes a method for calculating the beam grazing angle of SBR using coordinate transformation techniques. The method is characterized by its clear process and simple calculation. The effectiveness of this method has been verified through simulation experiments, providing a practical method for the real-time calculation of the beam grazing angle of SBR.

## Introduction

The beam grazing angle of SBR systems specifically refers to the angle between the radar beam and the Earth’s local horizontal plane, which directly impacts the radar’s detection accuracy and range. To optimize the performance of the radar system, accurate calculation of the beam grazing angle is particularly important. However, due to the dynamic motion of the satellite platform, the curvature of the Earth, and the effect of the Earth’s rotation, this calculation process becomes particularly complex and challenging. Although the reference^5^ discusses sea clutter characteristics and target detection techniques, it does not adequately address the SBR beam grazing angle calculation strategies, lacking a specific calculation framework and algorithm analysis. Lin and Wu^3^,, Pillai^6^,, while providing theoretical estimation methods and geometric correlations for the beam grazing angle, the motion factors of the satellite platform are not considered.

Given this context, based on SBR’s low-earth orbit satellite platform, this paper introduces a clear and efficient method for calculating the beam grazing angle. It takes into account the Earth’s non-spherical gravitational perturbations and atmospheric drag and incorporates the concept of coordinate transformation. Simulation experiments with STK (Systems Tool Kit) software have confirmed the feasibility and effectiveness of this method.

## The principle of beam grazing angle calculation

**Fig. 1:**
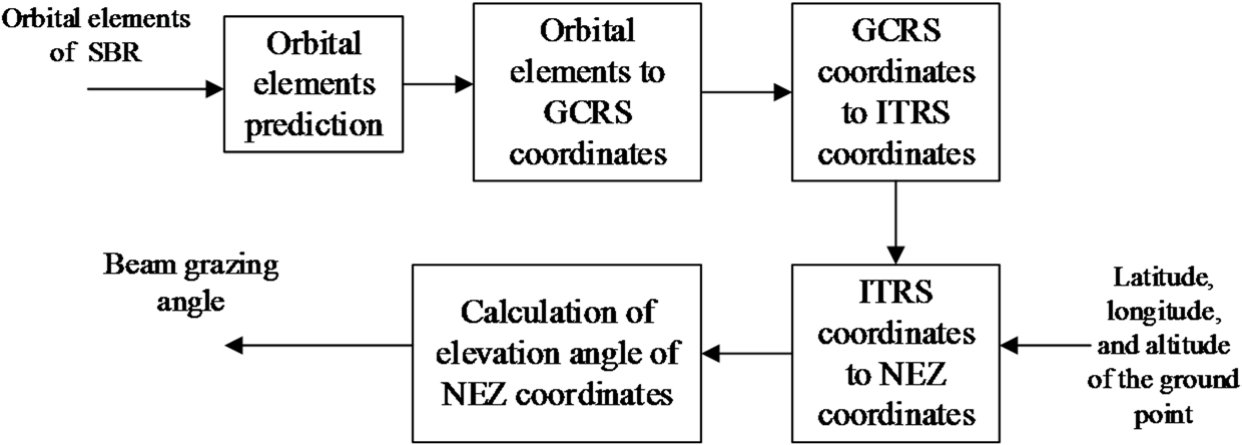
Flowchart for the calculation of SBR beam grazing angle.

The calculation process for the beam grazing angle of the SBR is shown in Fig. 1. This process uses the latitude, longitude, and altitude of a ground point, along with the SBR’s orbital elements at time *t*_0_, as inputs, and outputs of the beam grazing angle of the SBR at time *t*. First, based on the orbital elements of the SBR at time *t*_0_, orbit prediction is performed to obtain the orbital elements at time *t*. Then, the predicted orbital elements are converted into coordinates in the Geocentric Celestial Reference System (GCRS). Next, the GCRS coordinates are transformed into coordinates in the International Terrestrial Reference System (ITRS). Subsequently, the ITRS coordinates are transformed into a Topocentric-Horizon Coordinate System with the ground point as the origin, specifically using the North-East-Zenith (NEZ) Coordinate System. Finally, the elevation angle is calculated based on the cartesian coordinates in the NEZ system. For the SBR, this elevation angle corresponds to the beam grazing angle.

For concepts related to the coordinate systems mentioned above, please refer to^1^. The specific calculation principles will be detailed in the following sections.

### Calculation process of the beam grazing angle

**Orbital elements prediction.** The motion state of a satellite is typically described by six orbital elements^7^, including the semi-major axis *a*, eccentricity *e*, inclination *i*, right ascension of the ascending node *Ω*, argument of perigee *ω*, and true anomaly *f* (mean anomaly *M* is often used in practice instead), that is, (*a*, *e*, *i*, *Ω*, *ω*, *f* or *M*).

For low-earth orbit SBR, the main perturbations of its orbit are due to atmospheric drag and the non-spherical gravitational field of the Earth. This paper focuses on the long-term effects of these perturbing forces on the orbital elements and adopts a simplified analytical orbit prediction strategy: assuming the eccentricity remains constant; calculating the long-term variations of the right ascension of the ascending node and the argument of perigee using a simplified gravitational model (*J*_2_ term); and handling the cases with and without the mean motion derivative separately: if available, the long-term effects of atmospheric drag on the semi-major axis, eccentricity, and mean anomaly can be approximately calculated; if not, the effects of atmospheric drag are neglected. This method avoids complex atmospheric drag modeling and achieves a good balance between computational efficiency and prediction accuracy. The specific prediction formulas are shown as follows^8^:

1 $$\left\{ {\begin{array}{*{20}{l}} {a={a_0} - \frac{{2{a_0}}}{{3{n_0}}}{{\dot {n}}_0}\vartriangle t,}&{e={e_0} - \frac{{2(1 - {e_0})}}{{3{n_0}}}{{\dot {n}}_0}\vartriangle t} \\ {i={i_0},}&{\Omega ={\Omega _0} - \frac{{3{n_0}R_{{\mathrm{e}}}^{2}{J_2}}}{{2p_{0}^{2}}}\cos (i)\vartriangle t} \\ {\omega ={\omega _0}+\frac{{3{n_0}R_{{\mathrm{e}}}^{2}{J_2}}}{{4p_{0}^{2}}}\left[ {4 - 5{{\sin }^2}(i)} \right]\vartriangle t,}&{M={M_0}+{n_0}\vartriangle t+\frac{{{{\dot {n}}_0}}}{2}\vartriangle {t^2}+\frac{{{{\ddot {n}}_0}}}{6}\vartriangle {t^3}} \end{array}} \right.$$

where the subscript “0” denotes the initial time, *n*_0_ is the mean motion (${\dot {n}_0}$and ${\ddot {n}_0}$are its first and second derivatives, respectively), *R*_e_ is the semi-major axis of the Earth’s ellipsoid (i.e., the Earth’s equatorial radius, with a value of 6378.137 km), *J*_2_ is the zonal harmonic coefficient of the Earth’s gravitational field (with a value of 0.00108263), and Δ*t* is the orbital prediction time interval.

For LEO satellites at ~ 600 km altitude, perturbation magnitudes follow the hierarchy: J₂ effects ≫ Atmospheric Drag > High-order Geopotential Terms > Third-Body Gravitational Perturbations. This justifies the theoretical prioritization of J₂ and atmospheric drag modeling. While atmospheric drag effects are incorporated into the orbit propagation framework through the mean motion rate parameter (Eq. 1), the algorithm defaults to a J₂-only solution when mean motion rate parameters are unavailable.

High-order geopotential terms (e.g., J₂₂) exhibit magnitudes 2–3 orders smaller than J₂ (10⁻³–10⁻⁴ relative to J₂), justifying their exclusion. A high-precision atmospheric density model was not employed because empirical solar activity models introduce significant uncertainties in density predictions, which could degrade operational robustness. The proposed approach achieves an optimal balance between prediction accuracy and computational efficiency by estimating the mean motion derivative through orbit determination processes.

**Fig. 2:**
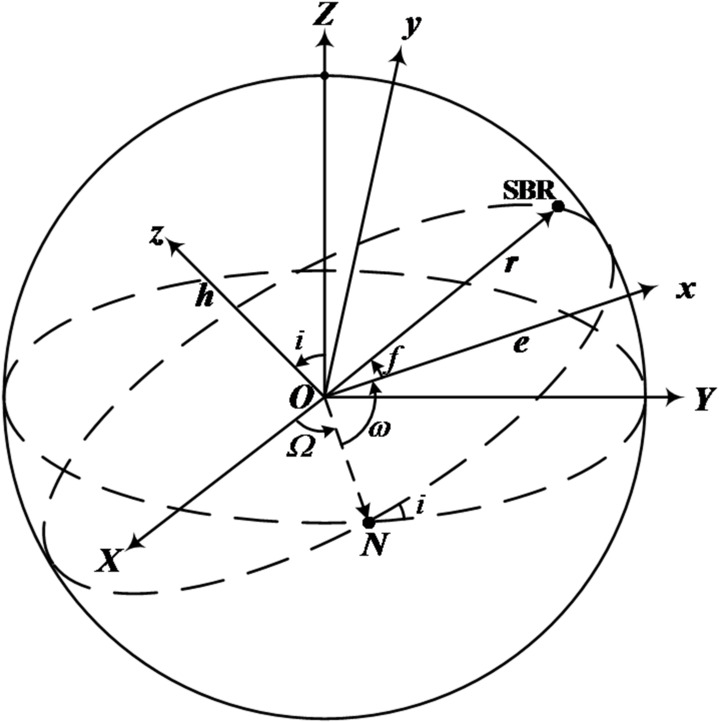
Diagram of transformation from orbital coordinate system to GCRS.

**Orbital elements to GCRS coordinates.** In Fig. 2, O-*XYZ* represents the GCRS, and *O*-*xyz* represents the orbital coordinate system.

According to the principles of coordinate transformation^1^, given the orbital elements of the SBR, the position vector in the GCRS is:

2 $${{\mathbf{r}}_{{\mathrm{GCRS}}}}={{\mathbf{R}}_3}( - \varOmega ){{\mathbf{R}}_1}( - i){{\mathbf{R}}_3}( - \omega )\left[ {\begin{array}{*{20}{c}} {r\cos f} \\ {r\sin f} \\ 0 \end{array}} \right]=r\cos f \cdot {\mathbf{P}}+r\sin f \cdot {\mathbf{Q}}$$

where ***r***_GCRS_ is the SBR’s position vector in GCRS, *r* = *a*(1-*e*^2^)(1 + *e*cos*f*)^−1^ is the geocentric distance of the SBR, *p* = *a*(1-*e*^2^) is the semi-latus rectum of the elliptical orbit, *h*=(*pµ*)^1/2^ is the specific angular momentum of the satellite’s orbit, *µ* is the Earth’s gravitational constant. The unit vectors ***P*** and ***Q*** of the *x*- and *y*-axes in the *O*-*XYZ* coordinate system, respectively, can be expressed as follows^1^:

3 $${\mathbf{P}}=\left[ {\begin{array}{*{20}{c}} {\cos \varOmega \cos \omega - \sin \varOmega sin\omega \cos i} \\ {sin\varOmega \cos \omega +\cos \varOmega sin\omega \cos i} \\ {sin\omega \sin i} \end{array}} \right],\quad {\mathbf{Q}}=\left[ {\begin{array}{*{20}{c}} { - \cos \varOmega sin\omega - \sin \varOmega cos\omega \cos i} \\ { - sin\varOmega sin\omega +\cos \varOmega cos\omega \cos i} \\ {cos\omega \sin i} \end{array}} \right]$$

### GCRS coordinates to ITRS coordinates

**Fig. 3:**
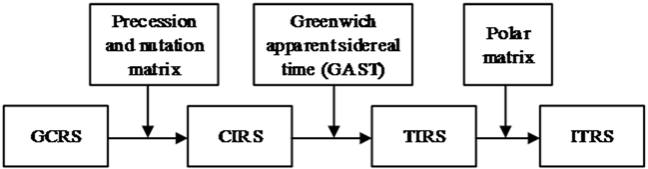
Flowchart of the transformation from GCRS to ITRS (based on the “IAU 2006/2000A-CIO” standard).

Figure 3 illustrates the transformation process between the GCRS and ITRS based on a non-rotating origin^2,4^. The transformation involves two intermediate reference systems: the Celestial Intermediate Reference System (CIRS) and the Terrestrial Intermediate Reference System (TIRS), as well as relevant astronomical parameters. The transformation formulas between them are:

4 $${{\mathbf{r}}_{{\mathrm{ITRS}}}}={\mathbf{C}}_{{{\mathrm{GCRS}}}}^{{{\mathrm{ITRS}}}} \cdot {{\mathbf{r}}_{{\mathrm{GCRS}}}}$$

where ***r***_ITRS_ is the SBR’s position vector in ITRS, ${\mathbf{C}}_{{{\mathrm{GCRS}}}}^{{{\mathrm{ITRS}}}}$representing the coordinate transformation matrix from GCRS to ITRS.

### ITRS coordinates to NEZ coordinates

**Fig. 4:**
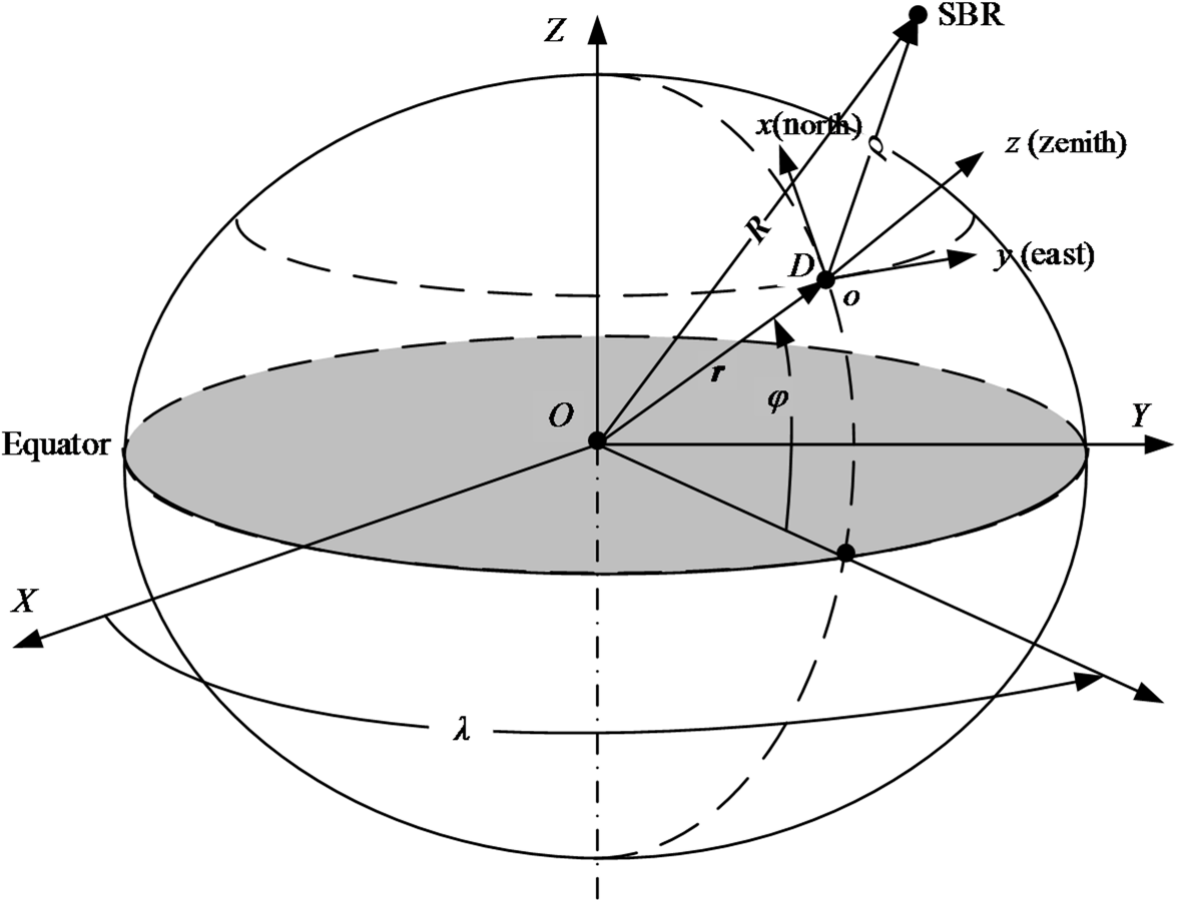
Diagram of transformation from ITRS to NEZ.

In Fig. 4, O-*XYZ* represents the ITRS, and *O*-*xyz* represents the NEZ. The transformation between them is given by^1^:

5 $${{\mathbf{r}}_{{\mathrm{NEZ}}}}={{\mathbf{P}}_1}{{\mathbf{R}}_2}({90^ \circ } - \varphi ){{\mathbf{R}}_3}(\lambda )({{\mathbf{r}}_{{\mathrm{ITRS}}}} - {{\mathbf{r}}_{{\mathrm{D,ITRS}}}})$$

where ***P***_1_=diag[−1, 1, 1], *λ* and *ϕ* are the astronomical longitude and latitude of ground point *D*. ***r***_D, ITRS_ represents the cartesian coordinates of ground point *D* in the ITRS, which are usually calculated from the geodetic coordinates of point *D* (longitude *L*, latitude *B*, and height *H*)^1^:

6 $$\left\{ \begin{gathered} X=(N+H)\cos B\cos L \hfill \\ Y=(N+H)\cos B\sin L \hfill \\ Z=[N(1 - {e^2})+H]\sin B \hfill \\ \end{gathered} \right.$$

where *N* = *R*_e_(1-*e*^2^sin^2^*B*)^−1/2^ is the radius of curvature in the prime vertical, *e* is the first eccentricity of the ellipsoid.

### Elevation angle calculation

**Fig. 5:**
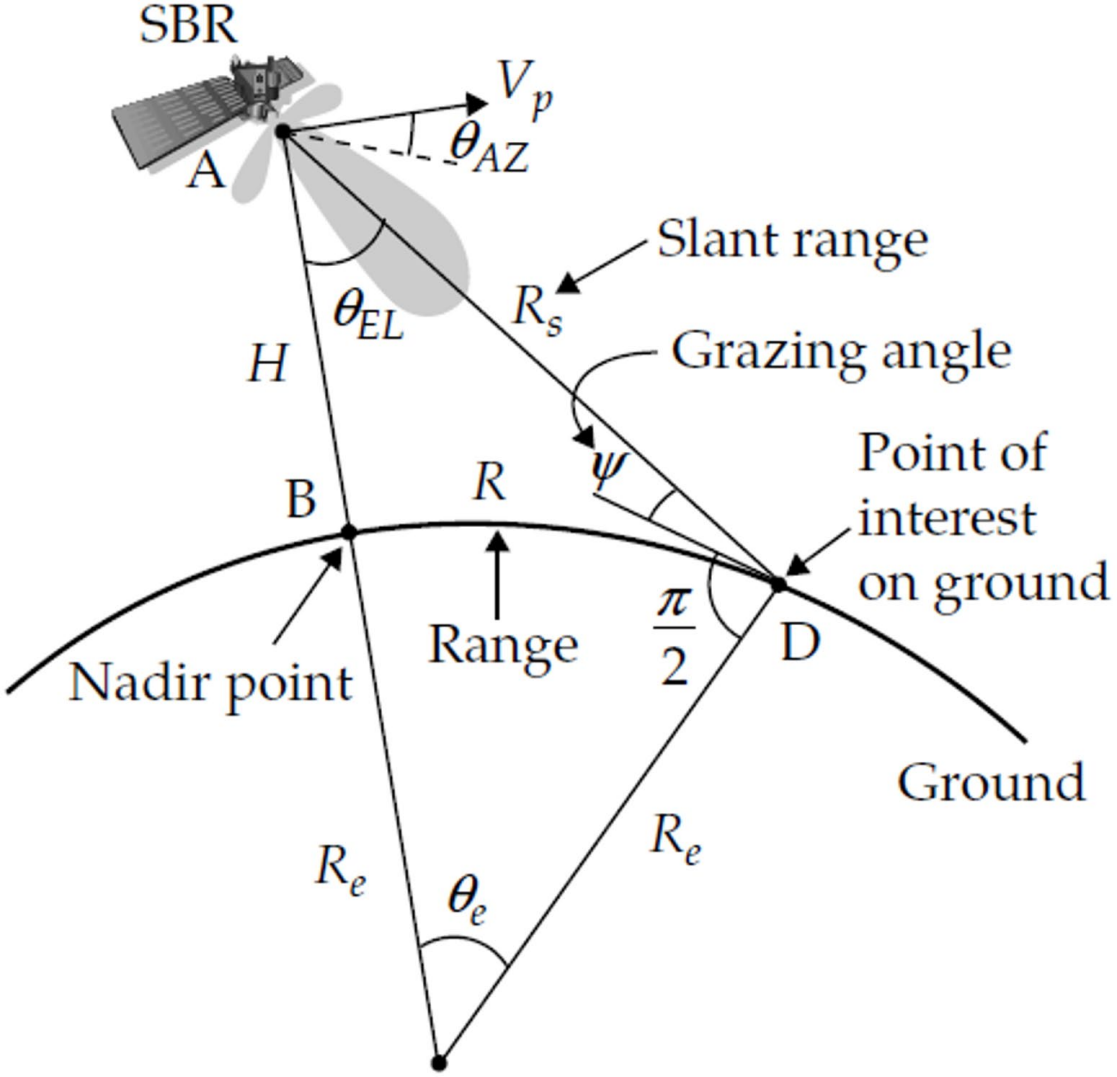
Diagram of the beam grazing angle of the SBR.

In Fig. 5, *ψ* represents the beam grazing angle of the SBR relative to the ground point *D*. If we establish an NEZ coordinate system with *D* as the observation station and SBR as the observed target, then *ψ* is equivalent to the elevation angle when observing SBR from point *D*. Assuming that the cartesian coordinates of SBR in the NEZ coordinate system are (*x*, *y*, *z*), the formula for calculating *ψ* is as follows.

7 $$\psi =\arctan \frac{z}{{\sqrt {{x^2}+{y^2}} }}$$

## Simulation experiments

The STARLINK-32,784 satellite is used as the satellite platform for simulating the SBR, and its TLE data is obtained from the SpaceTrack website. The main parameters extracted from the original data are shown in Table 1. The semi-major axis of the satellite can be calculated using the mean motion *n* through the formula *n*^2^*a*^3^ = *µ*.

**Table 1:** Orbital parameters of the STARLINK-32,784 satellite.

Item	Value
Observation epoch	2025-01-24 08:00:01 UTC
Mean motion derivative (rev/day^2^/2)	−0.0119077
Mean motion second derivative (rev/day^3^/6)	0
Inclination (deg)	43.0063
Right ascension of the node (deg)	73.5542
Eccentricity	0.0001327
Arg of perigee (deg)	278.729
Mean anomaly (deg)	276.7708
Mean motion (rev/day)	15.78322176

The validity of the method presented in this paper is verified using STK software. The problem description is as follows.

**GIVEN**: The TLE data of STARLINK-32,784 at time *t*_0_ (2025-01-24 08:00:01) and the geodetic coordinates of ground point *D* (120°, 30°, 0).

**FIND**: Analyze the impact of orbital perturbations on the radial velocity of ground point *D*. Calculate the beam grazing angle *ψ* of the SBR relative to point *D* at time *t* (2025-01-24 21:13:20).

Based on the description in Sect. 2, the detailed steps of the beam grazing angle calculation method presented in this paper are as follows:

(1) Parse the TLE data for STARLINK-32,784 to obtain various parameters of the satellite shown in Table 1;

(2) Use Eq. (1) to calculate the orbital elements of the SBR at time *t:*

(3) Use Eq. (2) to calculate the GCRS coordinates of the SBR;

(4) Use Eq. (4) to calculate the ITRS coordinates of the SBR;

(5) First, calculate the ITRS coordinates of ground point *D* based on its geodetic coordinates using Eq. (6), and then use Eq. (5) to calculate the NEZ coordinates of the SBR;

(6) Use Eq. (7) to calculate the beam grazing angle.

The steps for constructing the STK scenario are as follows:

(1) Set the start time of the STK scenario *t*_0_ and the end time to be longer than the solution time *t*, such as 2025-01-25 08:00:00;

(2) Create a new satellite (Satellite) in STK using the SGP4 propagation model and input the original TLE data as the orbital elements of the satellite;

(3) Create a new ground point (Place) in STK and input its geodetic coordinates;

(4) Use the Access tool in STK to calculate the elevation angle of the satellite from the ground point:

(5) Use the STK reporting function to output the beam grazing angle at time *t*.

The beam grazing angle calculated by our method at 2025-01-24 21:13:20 is 25.504°, identical to the result of 25.504° output by STK. Furthermore, we calculated the beam grazing angle at 10-second intervals between 2025-01-24 09:42:00 and 2025-01-24 09:47:40, totaling 35 data points. Figure 5 shows the comparison results, which demonstrate that the calculated results are consistent.

**Fig. 6:**
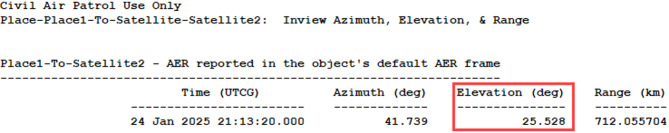
The beam grazing angle output by STK at time *t* (2025-01-24 21:13:20).

**Fig. 7:**
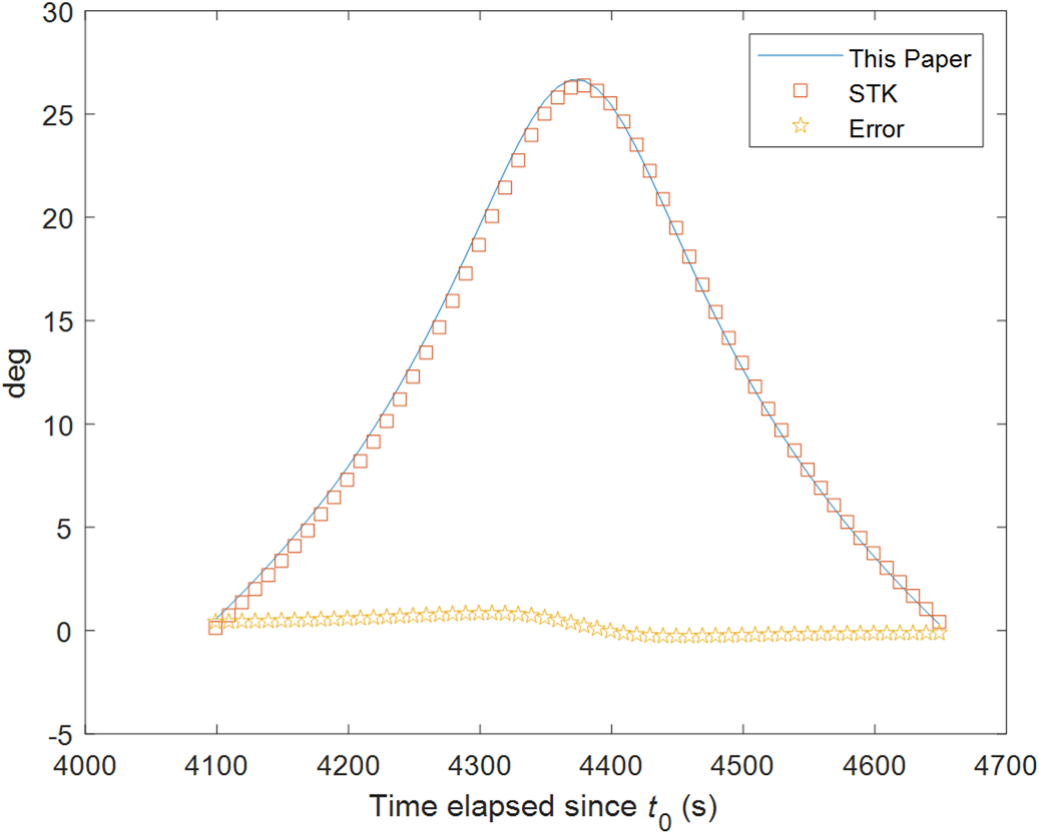
Comparison of beam grazing angles between STK and our method.

The elevation angle calculated by our method at 2025-01-24 21:13:20 is 25.504°, while the output from STK is 25.528°, as shown in Fig. 6. The error is very small, only 0.093%. Furthermore, the beam grazing angle was calculated at 10-second intervals from 2025-01-24 21:08:20 to 2025-01-24 21:17:30, resulting in a total of 56 data points. Figure 7 presents a comparison chart, which shows that the results from both methods are generally consistent.

The 0.093% discrepancy mainly stems from GCRS-to-ITRS coordinate transformation, driven by:

(1) EOP data recency differences – our study used IERS eopc04 (2023), while STK 12.2 used EOP-All (2021);

(2) Interpolation divergence – we applied 4th-order Lagrange to all EOPs, whereas STK employs 8th-order Lagrange for polar motion, cubic spline for UT1, and linear for nutation;

(3) Computational precision – our MATLAB-based double-precision implementation vs. STK’s C + + extended-precision computation.

## Conclusion

Based on the perturbed motion model of a low-earth orbit satellite platform, this paper proposes a method for calculating the beam grazing angle using the concept of coordinate transformation. The method is conceptually clear, and its effectiveness is verified through STK simulations.

The precision of GCRS-to-ITRS coordinate transformations is constrained by five primary factors: (1) Framework selection: CIP-based models demonstrate superior accuracy compared to equinox-based formulations; (2) Precession-Nutation model complexity: the 1976/1980 framework offers 0.1 arcsec precision with high efficiency for legacy systems, the 2006/2000A model achieves microarcsecond accuracy via 1066-term series but at low operational efficiency, while the 2000B compromise model balances 0.01 arcsec accuracy (77 terms) with moderate computational demands; (3) EOP interpolation: In-range interpolation typically introduces ≈ 0.1 milliarcsecond (mas) errors, whereas extrapolation beyond observed data ranges results in degraded accuracy (1–10 mas); (4) Computational trade-offs: While spline or Lagrange interpolation methods enhance numerical precision, they impose higher computational loads relative to simplified linear interpolation approaches. (5) EOP parameter corrections: the uncorrected method maintains a relatively high baseline error with lower computational demands, whereas ocean/solid-earth tide corrections significantly reduce error levels but require substantially more processing resources.

The computational accuracy of our method is primarily dependent on the precision of orbit propagation. Following orbital maneuvers of the space-based warning radar, the osculating orbital elements must be determined promptly and accurately; failure to do so will introduce significant propagation errors, resulting in miscalculation of the beam grazing angle.

Future research will explore the clutter Doppler characteristics of SBR under perturbed motion conditions. The coordinate transformation-based method proposed in this paper can not only calculate the angular information of the target relative to the radar (such as the beam grazing angle), but also determine the target’s velocity relative to the radar in the topocentric horizon coordinate system, which is the radial velocity. This radial velocity can then be used to compute the Doppler frequency.

## Data Availability

The authors confirm that all experimental data are available and accessible via the main text and/or the supplemental data.

## References

[1] Huang, X. B., Xiao, R. & Zhang, Y. *Fundamental Theory and Simulation Application of Orbital Space Objects* (Huazhong University of Science and Technology, 2024).

[2] Lei, W. W. & Zhang, H. W. A reference system transformation method based on the non-rotating origin and its computation. *Journal of Spacecraft TT&C Technology* **35**(4), 276–285 (2016).

[3] Lin, Y. & Wu, N. *Space-based Early Warning Radar* (National Defence Industry, 2018).

[4] Liu, L. & Hou, X. Y. *Fundamentals of Orbital Mechanics* (Higher Education Press, 2018).

[5] Liu, N. B., Jiang, X. Y., Ding, H. & Guan, J. Summary of Research on Characteristics of Radar Sea Clutter and Target Detection at High Grazing Angles. *J. Electron. Inform. Technol.* **43** (10), 2771–2780 (2021).

[6] Pillai, S., Unnikrishna, Li, K. & Himed, B. *Space-based Radar Theory and Applications* (House of Electronics Industry, 2016).

[7] Sellers, J. J., Astore, W. J. & Giffen, R. B. *W. J. Larson, Understanding Space: An Introduction to Astronautics* 3rd edn (McGraw-Hill College, 2005).

[8] Vallado, D. A. *Fundamentals of Astrodynamics and Applications* 5th edn (McGraw-Hill, 2023).

